# Strophanthus and Strophanthin

**Published:** 1907-06-22

**Authors:** 


### Points in Therapeutics.

### STROPHANTHUS AND STROPHANTHIN.

We have already discussed the variability in the strengths of tinctures of digitalis obtained from various sources, and have pointed out how necessary it is to have digitalis standardised physiologically in order that we may always be sure that the preparation we are employing in a particular case of heart disease is really active. This necessity for physiological standardisation depends upon the active principle being, not an alkaloid, but a glucoside which decomposes when any attempt is made to estimate it chemically. The same applies to the active principle of strophanthus, and it seems likely that the disrepute of strophanthus as a cardiac stimulant largely depends upon lack of activity in the preparations employed. There are various kinds of crude strophanthus, some better than others; the following are some interesting experiments carried out in the scientific laboratories that are supported by Messrs. Southall Bros. and Barclay in Birmingham. Ten years ago the late Mr. John Barclay described a method for the assay of tincture of strophanthus based upon the determination of the strophanthidin yielded by the hydrolysis of the strophanthin present. This method he extended at a later date to the extract introduced by the compilers of the 1898 Pharmacopœia. The process was expeditious, and gave concordant results under similar conditions; indeed, Cæsar and Loretz in 1905 published a method, the essential principle of which is identical, but which is vitiated by the introduction of hydrogen sulphide to remove the excess of lead salts, with the result that, as Fraser proves, much of the strophanthin is decomposed. It has always been felt that a method involving the direct determination of the active principle, and not of a decomposition product, is required; for, in addition to other objections, there is almost a certainty that the evaporation of an aqueous solution of strophanthin in contact with the organic acids of the seeds leads to partial hydrolysis. It has also been felt that, unless the seeds used in the preparation of the tincture had been accurately verified, the determination of strophanthidin would afford no criterion of the therapeutical value of the tincture.

### The Method of Examination.

The method of examination which was tried experimentally was as follows:—100 grams of the seeds were powdered and exhausted with petroleum spirit; the oil was separated and tested. The oil-free powder was air dried, transferred to a Dreschel extractor, and percolated for 30 hours with boiling absolute alcohol; the percolate was evaporated, and the residue, when cold, taken up with water. To this aqueous liquid a slight excess of solution of basic lead acetate was added, the liquid filtered, the filtrate treated with excess of sodium sulphate, filtered, and the resultant filtrate evaporated at a low temperature with 10 grams of fine sand. The product was powdered and exhausted in a Soxhlet tube with boiling amyl alcohol; the bulk of the solvent was removed on a water bath and evaporation completed at 60° C.

Various samples of crude strophanthus were treated in this way, namely: —

1. Seeds from a commercial parcel, origin unknown, but giving a green reaction with 80 per cent. sulphuric acid.
2. Seeds of *Strophanthus Kombé*, "Mandala brand," giving green reaction.
3. Seeds of *Strophanthus Nicholsoni*, verified. These seeds gave a distinct red reaction with the acid test.
4. Seeds of *Strophanthus gratus*, which were guaranteed authentic and gave a yellowish pink colour with the acid test.

Both the oils and the "strophanthins" obtained from these were analysed and tested.

As regards the oils, there were no particular differences in the four: —

| | Seeds of uncertain source. | Seeds of Strophanthus Kombé, Mandala. | Seeds of Strophanthus Nicholsoni. | Seeds of Strophanthus gratus. |
|---|---|---|---|---|
| Percentage of oil in the seeds | 34.08 | 34.76 | 20.90 | 35.01 |
| Specific gravity of the oil ... | 0.9249 | 0.9278 | 0.9219 | 0.9230 |
| Free acid, calculated as oleic acid, per cent. ... ... ... | 7.55 | 6.84 | 14.84 | 5.17 |
| Saponification number ... ... | 192.0 | 189.7 | 190.5 | 191.3 |
| Iodine absorbed in 18 hours, per cent. ... ... ... ... | 100.7 | 99.4 | 99.7 | 93.9 |
| Melting-point of fatty acids ... | 33° C. | 33° C. | 33° C. | 29° C. |

The strophanthin obtained by the method described above was in each case crystalline, slightly reddish in colour, and highly deliquescent; recrystallised from amylic alcohol, long colourless needle crystals were obtained which gave highly characteristic colour reactions with sulphuric acid. Each specimen of strophanthin proved to be very slightly dextro-rotatory in alcoholic solution, while in no case could a sharp melting point be obtained from the anhydrous glucoside.

The peculiarity of behaviour in this respect is also marked when the results of testing physiologically are considered. The physiological tests were undertaken by Professor R. F. C. Leith, M.A., M.B., F.R.C.P., of Birmingham University, and for this purpose crystallised specimens of strophanthin were prepared by the above method from three varieties of seeds. The strophanthin was made up into a solution in 70 per cent. alcohol, representing 0.2 gram of the glucoside in 100 mils., the strength being fixed at this in order to approximately represent the tincture in strength as assayed by the strophanthidin method.

### Some General Conclusions.

Professor Leith conducted his experiments upon frogs, and his results are briefly as follows: —

Using solutions of the above strength and diluting the dose with normal saline before injection, the strophanthin obtained from *Strophanthus Nicholsoni* seeds produced no effect in doses of ¼ and ¾ minim; 1 minim slightly quickened the rate of respiration; 1½ minim had no further effect; all the frogs were alive and apparently well some weeks after the injection.

Using strophanthin obtained from the seeds of *Strophanthus Kombé, Mandala,* no apparent effect was produced by ¼ minim dose; ¾ minim caused rapid breathing; whilst 1 minim caused signs of illness, and 1½ minim was sufficient to cause death within an hour.

Strophanthin obtained from *Strophanthus gratus* seeds caused no apparent illness in ¼ and ¾ minim doses, but a 1 minim dose sufficed to kill a large and active frog within three hours; whilst 1½ minims destroyed life in a similar frog within 65 minutes.

The minimum lethal dose per 100 grams of frog, is therefore as follows:—

| | |
|---|---|
| Strophanthin from *Strophanthus Nicholsoni* ... | Not within the limits of the experiments |
| ,, ,, *Strophanthus Kombé* ... | 5 minims |
| ,, ,, *Strophanthus gratus* ... | 3·8 minims |

Continental workers have testified to the activity of the strophanthin derived from *Strophanthus gratus*, and these results bear out their opinions. Strophanthin from *Strophanthus Kombé, Mandala,* is not far behind in its toxicity, whilst that from *Strophanthus Nicholsoni* is comparatively feeble, or even inert.

Professor Dr. Feist has also shown that the strophanthin characteristic of the species *Strophanthus Kombé* and *Strophanthus gratus* are essentially different in chemical constitution, and he has even found yet other kinds of strophanthins. He considers that strophanthin derived from *Strophanthus gratus* is identical with Arnaud's oüabain, and that this name should be retained for it to prevent confusion. We cannot in the absence of more complete investigation subscribe unreservedly to Dr. Feist's conclusions, but we think that the results obtained without doubt support his view that the different varieties of strophanthus contain glucosides essentially different in their toxic effect, and, as judged by the "strophanthin" yield, different in chemical constitution.

Considering the whole question, these results appear to point to the possibility of chemically standardising strophanthus and its preparations, but at the same time to the fact that such standardisation can only have a real value when the botanical character of the seeds used is fully established; failing the latter, the activity of the drug can only be tested by physiological experiments. We are also confronted by the fact that the active glucoside principle in *Strophanthus gratus* exhibits decided variation from that of the officinal variety, and may not be strictly comparable in its physiological effects upon human beings.

If this article cannot by itself be converted to direct account by the busy medical man, it will at least serve to show how many things must be taken into account before such a preparation as tincture of strophanthus is at once branded as almost useless; it will also help to indicate how much work is being done nowadays in scientific laboratories, in the direction of placing therapeutics upon a scientific basis which can be understood.

